# Range of Motion and Neutral Zone of All Human Spinal Motion Segments: A Data Collection of 30 Years of In Vitro Experiments Performed Under Standardized Testing Conditions

**DOI:** 10.1002/jsp2.70052

**Published:** 2025-03-05

**Authors:** Hans‐Joachim Wilke, Annette Kienle, Karin Werner, Christian Liebsch

**Affiliations:** ^1^ Institute of Orthopaedic Research and Biomechanics, Trauma Research Centre Ulm Ulm University Medical Centre Ulm Germany; ^2^ SpineServ GmbH & Co. KG Ulm Germany

**Keywords:** biomechanics, cervical, flexibility, human, in vitro, lumbar, neutral zone, range of motion, spine, thoracic

## Abstract

**Background:**

Spinal flexibility can vary among spinal sections and single motion segments. The purpose of this work was to provide a comprehensive overview of the range of motion (RoM) and neutral zone (NZ) values of all spinal levels collected during 30 years of in vitro experiments under standardized testing conditions.

**Methods:**

RoM and NZ data obtained from in vitro testing of intact human mono‐ and polysegmental specimens with pure moments of 2.5 Nm for the cervical, 5 Nm for the thoracic, and 7.5 Nm for the lumbar spine were collated from the internal database of the authors' institution. Descriptive statistics were performed with median values and median absolute deviations. Outliers were defined as values beyond twofold standard deviation and excluded from evaluation. Normal distribution was verified using the Shapiro–Wilk test.

**Results:**

RoM and NZ data of *N* = 1139 functional spinal units were collected with sample sizes ranging from *n* = 6 to *n* = 224 per segmental level. The cervical spine was very flexible in flexion/extension and moderately flexible in lateral bending, while in axial rotation, the motion segment C1‐C2 was as flexible as the subaxial cervical spine combined. The thoracic spine was the least flexible section in flexion/extension but allowed moderate lateral bending and axial rotation. The rib cage had a strong effect on thoracic spinal flexibility, particularly in axial rotation and at the mid‐thoracic spinal levels. The lumbar spine exhibited moderate flexibility in flexion/extension and lateral bending but showed the lowest RoM and NZ in axial rotation.

**Conclusions:**

This unique summary of RoM and NZ data, acquired under the same loading conditions in the same spine tester, provides a detailed insight into overall spinal flexibility and will serve as a valid dataset for the validation of in vitro studies and numerical models of the single motion segments of the spine.

## Graphical abstract

A comprehensive overview of the range of motion (RoM) and neutral zone (NZ) values of all spinal levels is presented, which were collected during 30 years of in vitro experiments under standardized testing conditions in one institution. This unique summary of RoM and NZ data, acquired under the same loading conditions in the same testing device, provides a detailed insight into overall spinal flexibility and will serve as a valid dataset for the validation of in vitro studies and numerical models of the single motion segments of the spine.

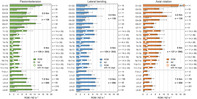

## 1 Introduction

Range of motion and neutral zone represent fundamental parameters for the quantification of spinal flexibility and are essential for the interpretation of the effects of pathological changes and surgical treatments on spinal stability. Both parameters are known to vary among spinal regions but also vary from motion segment to motion segment within these regions. While numerous in vivo, in vitro, and in silico studies have reported on segmental range of motion and neutral zone values of the human spine, literature data exhibit large variations due to interindividual differences of the investigated motion segments regarding morphology and material properties caused by various natural factors such as aging and degeneration [1]. Apart from that, spinal flexibility is highly dependent on the individual movement comfort and body posture of participants as well as the measurement technique when being quantified in vivo [2, 3, 4, 5, 6, 7]. In contrast, in vitro studies provide the major benefit of direct measurement of spinal motions under controlled loading. However, experimental designs and testing conditions of in vitro setups, particularly the type and level of loading, can vary considerably between single studies and thus also influence the resulting flexibility [8, 9, 10, 11, 12]. Moreover, the vast majority of in vitro studies on spinal ranges of motion and neutral zones are performed on single segmental levels or specific spinal sections, limiting data comparability and interpretability in the case of polysegmental investigations across multiple spinal sections, and use different load application methods and motion measurement techniques. Therefore, compilations of segmental range of motion and neutral zone data of the entire spine acquired under standardized and thus reproducible testing conditions are required to enhance literature comparisons and to improve the quality of in vitro and in silico model validations.

White and Panjabi were the first who published a comprehensive overview of segmental ranges of motion of the whole spine in flexion/extension, lateral bending, and axial rotation [13, 14], showing that spinal flexibility considerably varies between segmental levels. Ever since, this extensive summary of range of motion data has been widely used for literature comparisons and has served as input and validation data for multiple modeling studies. However, White and Panjabi's overview presents data of which “the authors consider (…) to be the most representative values for rotatory ranges of motion” [14], combining data of multiple sources “based on careful review of the literature” [14] comprising different study designs and testing conditions and neither reporting on ranges of variations, nor on effects of potential influencing factors such as age, nor on neutral zone data. Nevertheless, White and Panjabi's overview is still frequently used for model validation purposes, as there are only few comprehensive overviews of spinal range of motion and neutral zone in literature, which are furthermore primarily focused on specific spinal sections [8, 9, 10, 11, 12].

The purpose of the present work, therefore, was to collate and evaluate the range of motion and neutral zone data for each motion segment of the entire human spine that were acquired from in vitro experiments over more than 30 years at one single institution under standardized in vitro testing conditions in order to provide a comprehensive overview of human spinal flexibility.

## 2 Materials and Methods

### 2.1 Inclusion Criteria

For this database evaluation, any range of motion and neutral zone data of the authors' institution were included, which originated from (1) in vitro testing of (2) human (3) intact spine specimens under the application of (4) pure moment loading in the (5) three principal motion planes: flexion/extension, lateral bending, and/or axial rotation. The intact condition of specimens was defined as the preservation of all biomechanically relevant spinal structures, that is, vertebrae, discs, ligaments, and facet capsules, of the respective functional spinal units.

### 2.2 Flexibility Testing

**FIGURE 1:**
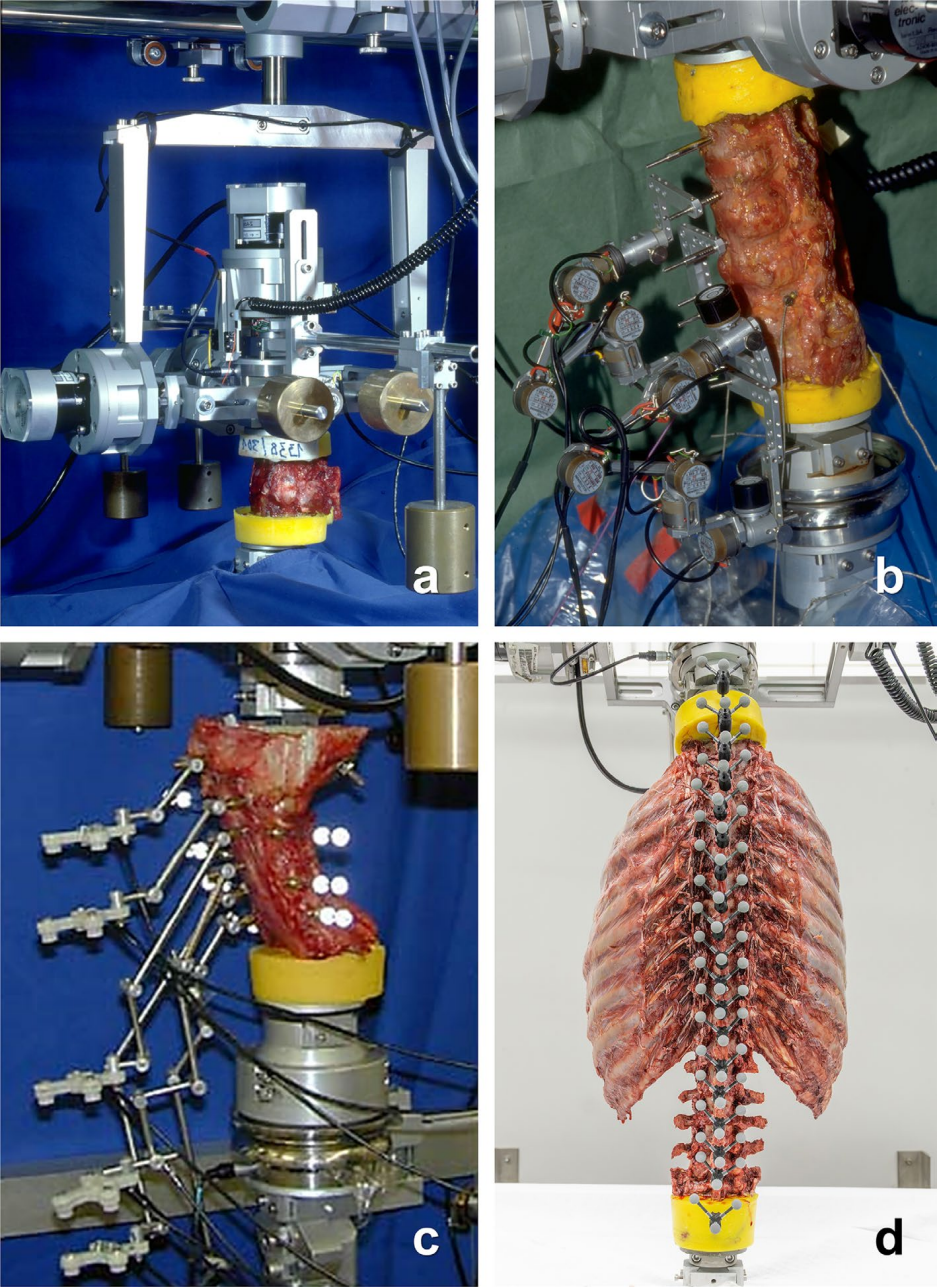
Measurement systems used in studies at the authors' institution between 1995 and 2024: Rotary variable displacement transducers integrated into the cardan joint of the spine tester (a), three‐dimensional goniometric linkage system (b), ultrasound‐based motion analysis system Zebris (c), optical motion tracking system Vicon MX13 (d).

All evaluated flexibility measurements were performed in an established custom‐made spine tester [15] under the same standardized testing conditions [16] without axial preload and with the five uncontrolled degrees of freedom being unconstrained. Unconstrained movement was allowed by a gimbal system integrated into a traveling gantry. The gimbal system was equipped with stepper motors, which could be engaged and driven separately, as well as balancing weights to ensure loading with pure moments in the six degrees of freedom. For testing, the caudal end of each specimen was rigidly fixed to the base of the spine tester and the cranial end to the gimbal system. All specimens underwent quasi‐static loading with a constant angular velocity between 0.5°/s and 1°/s for 3.5 load cycles, of which the first two cycles served for preconditioning and the third full cycle for data evaluation in order to minimize viscoelastic effects. Prior to loading, specimens were driven to a starting moment of 0 Nm. Moments were measured using a six‐component load cell (Schunk, Lauffen/Neckar, Germany) mounted between the gimbal system and the cranial end of the specimen. Load and displacement data were continuously recorded at a sampling rate of 50 Hz. The specimens were tested fully thawed at room temperature and were kept moist with saline solution to avoid dehydration during testing [17]. The upper‐ and lowermost vertebrae of each specimen were half embedded in polymethylmethacrylate (PMMA, Technovit 3040, Heraeus Kulzer, Wehrheim, Germany), on which metal flanges were screwed for rigid fixation in the spine tester. Tests were conducted on both mono‐ and polysegmental spine specimens, depending on the specific study design, while the flexibility data were evaluated for each segmental level in the case of polysegmental specimens. For the determination of the segmental ranges of motion and neutral zones, four different measurement systems were used within the 30 years of conducted experiments between 1995 and 2024 (Figure 1), all having similar precision of less than or equal to 0.1°:

- Rotary variable displacement transducers (RVDT, Novotechnik, Ostfildern, Germany; resolution 0.1°), which were integrated into the three axes of the gimbal system of the spine tester, respectively, allowed continuous recording of three‐dimensional rotary movements of monosegmental specimens.
- Three‐dimensional goniometric linkage system [18], consisting of six rotary angle potentiometers (P1701A502, Novotechnik, Ostfildern, Germany; resolution 0.1°), which were joined together by connecting elements in a way that the attached flange pieces could easily follow every movement. By combining multiple goniometer systems, monosegmental measurements in polysegmental specimens became feasible.
- High‐resolution ultrasound‐based motion analysis system (Zebris, Isny, Germany; sampling rate 20 Hz, resolution 0.1°), which enabled non‐contact measurements of three‐dimensional rotary movements of single motion segments in polysegmental specimens.
- Optical motion tracking system (Vicon MX13, Vicon Motion Systems Ltd., Oxford, UK; resolution 0.1°) consisting of six to twelve free‐standing infrared cameras. This marker‐based technique allowed non‐contact measurements of three‐dimensional monosegmental movements of polysegmental specimens in real time with three small markers per vertebra.

### 2.3 Data Processing and Statistics

**FIGURE 2:**
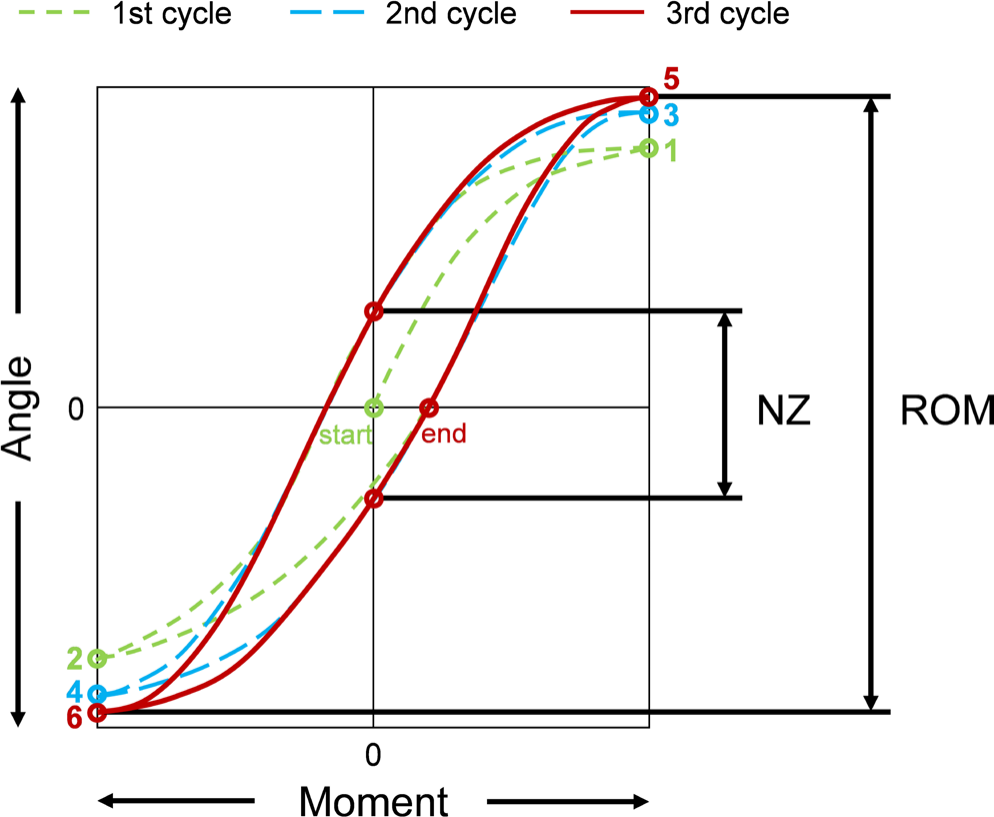
Schematic illustration of a typical angle‐moment hysteresis curve together with the evaluated parameters range of motion (RoM) and neutral zone (NZ). Modified according to Wilke et al. 1998 [16].

For the present overview of spinal flexibility data, the range of motion and neutral zone data were acquired from standard testing based on widely accepted recommendations for spinal in vitro testing [16] including pure moments of 2.5 Nm for the cervical spine, 5 Nm for the thoracic spine, and 7.5 Nm for the lumbar spine. Ranges of motion and neutral zones of the single motion segments were determined from the third loading cycles of the load‐deformation curves for the respective applied moments (Figure 2) using custom‐written Matlab scripts (MathWorks Inc. Natick, USA), automatically determining the zero points of the hysteresis curves by polynomial fitting and post‐processing using Excel (Microsoft Corp., Redmond, USA).

Descriptive statistics were performed by means of bar diagrams showing median values together with median absolute deviations of segmental range of motion and neutral zone for each level separately. Outliers were defined as values being beyond twofold standard deviation and excluded from the analysis. In order to prove data quality, normal distribution was verified using the Shapiro–Wilk test. Effects of donor age and sex, as well as the number of segments, on the range of motion and neutral zone were determined representatively for the level L4‐L5, which exhibited the largest sample sizes of all spinal levels, by means of linear regression analysis for age and the Mann–Whitney U test for sex and number of segments. Statistical evaluations were performed in SPSS29 (IBM Corp., Armonk, USA) with a significance level of 0.05.

### 2.4 Ethics

All data presented in this overview originate from studies that were conducted in accordance with the ethical principles of the Declarations of Helsinki. More recent studies (since 2012) were approved by the ethics committee of the University of Ulm (vote no. 383/12, 302/14, 35/16, 307/17, 487/17, 138/18, 332/19).

## 3 Results

### 3.1 Overall Data Collective

For this comprehensive overview, the range of motion and neutral zone data of *N* = 1139 human functional spinal units from C1‐C2 to L5‐S1 were collated by means of a database evaluation (Table 1). These data originated from 50 research projects and had been separately reported in 53 publications [17, 19, 20, 21, 22, 23, 24, 25, 26, 27, 28, 29, 30, 31, 32, 33, 34, 35, 36, 37, 38, 39, 40, 41, 42, 43, 44, 45, 46, 47, 48, 49, 50, 51, 52, 53, 54, 55, 56, 57, 58, 59, 60, 61, 62, 63, 64, 65, 66, 67, 68, 69, 70] (Tables 2, 3, 4).

**TABLE 1:** Overview of included data acquired from in vitro experiments using standard moments recommended by Wilke et al. (1998) [16]. *n* represents the total number of evaluated spinal levels (upper row) and the number of spinal levels from mono−/polysegmental specimens (lower row).

Spinal region	Applied moment (Nm)	Flexion/extension (*n*)	Lateral bending (*n*)	Axial rotation (*n*)	Age in years (median, range, mean ± SD)	Sex f/m
Cervical spine	2.5	138	139	136	63 (26–90)	67/72
(C1‐T1)		(75/63)	(68/71)	(77/59)	64 ± 14	
Thoracic spine	5.0	129	129	127	57 (37–80)	49/80
(T1‐T12)		(129/−)	(129/−)	(127/−)	56 ± 8	
Thoracic spine	5.0	253	253	286	55 (26–99)	176/110
(T1‐T12)		(−/253)	(−/253)	(−/286)	57 ± 18	
+rib cage						
Lumbar spine	7.5	590	596	590	57 (18–99)	215/345
(T12‐S1)		(308/282)	(309/287)	(290/300)	56 ± 16	(36 n.a.)
Whole spine	2.5, 5.0, 7.5	1110	1117	1139	57 (18–99)	507/607
(C1‐S1 + rib cage)		(513/597)	(506/611)	(494/645)	57 ± 15	(36 n.a.)

**TABLE 2:** Included studies on the cervical spine using pure moments of 2.5 Nm together with information on the number of tested specimens, the level(s) that were loaded in the spine tester, and the level(s) for which flexibility data was evaluated.

Reference	Publication title	Sample size	Tested level(s)	Evaluated level(s)
Pitzen et al. (1999) [21]	Evaluation of a new monocortical screw for anterior cervical fusion and plating by a combined biomechanical and clinical study	*n* = 12	C4‐C7	C5‐C6
Richter et al. (1999) [22]	Biomechanical evaluation of a newly developed monocortical expansion screw for use in anterior internal fixation of the cervical spine. In vitro comparison with two established internal fixation systems	*n* = 8	C4‐C7	C5‐C6
Richter et al. (2000a) [23]	Load–displacement properties of the normal and injured lower cervical spine in vitro	*n* = 6	C4‐C7	C5‐C6
Richter et al. (2000b) [26]	Biomechanical evaluation of a new modular rod‐screw implant system for posterior instrumentation of the occipito‐cervical spine: in‐vitro comparison with two established implant systems	*n* = 8	C0‐C5	C1‐C2 C2‐C3 C3‐C4 C4‐C5
Wilke et al. (2000a) [24]	Primary stabilizing effect of interbody fusion devices for the cervical spine: an in vitro comparison between three different cage types and bone cement	*n* = 8 *n* = 8 *n* = 8	C2‐C3 C4‐C5 C6‐C7	C2‐C3 C4‐C5 C6‐C7
Wilke et al. (2000b) [27]	Subsidence resulting from simulated postoperative neck movements: an in vitro investigation with a new cervical fusion cage	*n* = 8 *n* = 8 *n* = 8	C2‐C3 C4‐C5 C6‐C7	C2‐C3 C4‐C5 C6‐C7
Arand et al. (2002) [29]	The traumatic spondylolisthesis of the axis. A biomechanical in vitro evaluation of an instability model and clinical relevant constructs for stabilization	*n* = 6	C2‐C3	C2‐C3
Pitzen et al. (2007) [38]	Cervical spine disc prosthesis: radiographic, biomechanical and morphological post mortal findings 12 weeks after implantation. A retrieval example	*n* = 1	C3‐T1	C4‐C5 C5‐C6 C6‐C7
Schmieder et al. (2007) [37]	In vitro flexibility of the cervical spine after ventral uncoforaminotomy. Laboratory investigation	*n* =3 *n* = 3	C4‐C5 C6‐C7	C4‐C5 C6‐C7
Vogt et al. (2024) [69]	The move‐C cervical artificial disc can restore intact range of motion and 3‐D kinematics	*n* = 6	C4‐C5	C4‐C5

**TABLE 3:** Included studies on the thoracic spine using pure moments of 5 Nm, together with information on the number of tested specimens, the level(s) that were loaded in the spine tester, and the level(s) for which flexibility data was evaluated.

Reference	Publication title	Sample size	Tested level(s)	Evaluated level(s)
Liebsch et al. (2018) [55]	The effect of follower load on the intersegmental coupled motion characteristics of the human thoracic spine: An in vitro study using entire rib cage specimens	*n* = 8	C7‐L1 +rib cage	T1‐T2 T2‐T3 T3‐T4 T4‐T5 T5‐T6 T6‐T7 T7‐T8 T8‐T9 T9‐T10 T10‐T11 T11‐T12
Liebsch et al. (2019) [58]	In vitro analysis of kinematics and elastostatics of the human rib cage during thoracic spinal movement for the validation of numerical models	*n* = 8	C7‐L1 +rib cage	T1‐T2 T2‐T3 T3‐T4 T4‐T5 T5‐T6 T6‐T7 T7‐T8 T8‐T9 T9‐T10 T10‐T11 T11‐T12
Liebsch and Wilke (2020) [59]	Rib presence, anterior rib cage integrity, and segmental length affect the stability of the human thoracic spine: An in vitro study	*n* = 8	C7‐L1 +rib cage	T1‐T2 T3‐T4 T5‐T6 T7‐T8 T9‐10 T11‐T12
Liebsch et al. (2020a) [62]	In vitro comparison of personalized 3D printed versus standard expandable titanium vertebral body replacement implants in the mid‐thoracic spine using entire rib cage specimens	*n* = 6	C7‐L1 +rib cage	T1‐T2 T2‐T3 T3‐T4 T4‐T5 T5‐T6 T6‐T7 T7‐T8 T8‐T9 T9‐T10 T10‐T11 T11‐T12
Liebsch et al. (2020b) [63]	Thoracic spinal kinematics is affected by the grade of intervertebral disc degeneration, but not by the presence of the ribs: An in vitro study	*n* = 8 *n* = 8 *n* = 8 *n* = 8 *n* = 8 *n* = 8	T1‐T2 T3‐T4 T5‐T6 T7‐T8 T9‐T10 T11‐T12	T1‐T2 T3‐T4 T5‐T6 T7‐T8 T9‐T10 T11‐T12
Liebsch et al. (2020c) [64]	Thoracic spinal stability and motion behavior are affected by the length of posterior instrumentation after vertebral body replacement, but not by the surgical approach type: An in vitro study with entire rib cage specimens	*n* = 6	C7‐L1 +rib cage	T1‐T2 T2‐T3 T3‐T4 T4‐T5 T5‐T6 T6‐T7 T7‐T8 T8‐T9 T9‐T10 T10‐T11 T11‐T12
Liebsch and Wilke (2022) [68]	Even mild intervertebral disc degeneration reduces the flexibility of the thoracic spine: An experimental study on 95 human specimens	*n* = 11 *n* = 4 *n* = 13 *n* = 4 *n* = 11 *n* = 4 *n* = 13 *n* = 4 *n* = 14 *n* = 4 *n* = 13	T1‐T2 T2‐T3 T3‐T4 T4‐T5 T5‐T6 T6‐T7 T7‐T8 T8‐T9 T9‐T10 T10‐T11 T11‐T12	T1‐T2 T2‐T3 T3‐T4 T4‐T5 T5‐T6 T6‐T7 T7‐T8 T8‐T9 T9‐T10 T10‐T11 T11‐T12

**TABLE 4:** Included studies on the lumbar spine using pure moments of 7.5 Nm, together with information on the number of tested specimens, the level(s) that were loaded in the spine tester, and the level(s) for which flexibility data was evaluated.

Reference	Publication title	Sample size	Tested level(s)	Evaluated level(s)
Wilke et al. (1997) [19]	A mechanical model of human spinal motion segments	*n* = 6	L1‐S1	L4‐L5
Quint et al. (1998a) [20]	Laminectomy and functional impairment of the lumbar spine: The importance of muscle forces in flexible and rigid instrumented stabilization—a biomechanical study in vitro	*n* = 6	L2‐S2	L4‐L5
Quint et al. (1998b) [17]	Importance of the intersegmental trunk muscles for the stability of the lumbar spine. A biomechanical study in vitro	*n* = 18	L2‐S2	L3‐L4 L4‐L5
Kettler et al. (2000a) [25]	Stabilizing effect of posterior lumbar interbody fusion cages before and after cyclic loading	*n* = 12 *n* = 12	L2‐L3 L4‐L5	L2‐L3 L4‐L5
Rohlmann et al. (2001) [70]	Influence of a follower load on intradiscal pressure and intersegmental rotation of the lumbar spine	*n* = 10	L1‐L5	L1‐L2 L2‐L3 L3‐L4 L4‐L5
Wilke et al. (2001) [28]	Effect of a prosthetic disc nucleus on the mobility and disc height of the L4‐5 intervertebral disc postnucleotomy	*n* = 6	L4‐L5	L4‐L5
Wilke et al. (2003) [30]	ISSLS prize winner: A novel approach to determine trunk muscle forces during flexion and extension: A comparison of data from an in vitro experiment and in vivo measurements	*n* = 7	L1‐L5	L2‐L3 L4‐L5
Kettler et al. (2004) [31]	Finite helical axes of motion are a useful tool to describe the three‐dimensional in vitro kinematics of the intact, injured and stabilized spine	*n* = 11	L4‐L5	L4‐L5
Kettler et al. (2005) [33]	In vitro stabilizing effect of a transforaminal compared with two posterior lumbar interbody fusion cages	*n* = 12 *n* = 6	L2‐L3 L4‐L5	L2‐L3 L4‐L5
Wilke et al. (2005) [32]	Range of motion or finite helical axis? Comparison of different methods to describe spinal segmental motion in vitro	*n* = 11	L4‐L5	L4‐L5
Kettler et al. (2006) [34]	In vitro fixator rod loading after transforaminal compared to anterior lumbar interbody fusion	*n* = 6	L1‐L5	L1‐L2 L2‐L3 L3‐L4 L4‐L5
Niemeyer et al. (2006) [35]	In vitro study of biomechanical behavior of anterior and transforaminal lumbar interbody instrumentation techniques	*n* = 6	L1‐L5	L1‐L2 L2‐L3 L3‐L4 L4‐L5
Wilke et al. (2006) [36]	Biomechanical evaluation of a new total posterior‐element replacement system	*n* = 6	L3‐S1	L3‐L4 L4‐L5 L5‐S1
Heuer et al. (2007a) [39]	Stepwise reduction of functional spinal structures increases vertebral translation and intradiscal pressure	*n* = 8	L4‐L5	L4‐L5
Heuer et al. (2007b) [40]	Stepwise reduction of functional spinal structures increase range of motion and change lordosis angle	*n* = 8	L4‐L5	L4‐L5
Heuer et al. (2007c) [41]	Creep associated changes in intervertebral disc bulging obtained with a laser scanning device	*n* = 7	L4‐L5	L4‐L5
Heuer et al. (2008a) [45]	A new laser scanning technique for imaging intervertebral disc displacement and its application to modeling nucleotomy	*n* = 6	L4‐L5	L4‐L5
Heuer et al. (2008b) [46]	Stepwise reduction of functional spinal structures increase disc bulge and surface strains	*n* = 6	L2‐L3	L2‐L3
Kettler et al. (2008) [42]	Can a modified interspinous spacer prevent instability in axial rotation and lateral bending? A biomechanical in vitro study resulting in a new idea	*n* = 6 *n* = 6	L2‐L3 L4‐L5	L2‐L3 L4‐L5
Quint et al. (2008) [44]	Grading of degenerative disk disease and functional impairment: imaging versus patho‐anatomical findings	*n* = 18 *n* = 18	L3‐L4 L4‐L5	L3‐L4 L4‐L5
Wilke et al. (2008) [43]	Biomechanical effect of different lumbar interspinous implants on flexibility and intradiscal pressure	*n* = 12 *n* = 12	L2‐L3 L4‐L5	L2‐L3 L4‐L5
Wilke et al. (2009) [47]	Prospective design delineation and subsequent in vitro evaluation of a new posterior dynamic stabilization system	*n* = 6	L2‐L3	L2‐L3
Cakir et al. (2012) [48]	Resect or not to resect: The role of posterior longitudinal ligament in lumbar total disc replacement	*n* = 6	L2‐L3	L2‐L3
Heuer et al. (2012) [49]	Posterior motion preserving implants evaluated by means of intervertebral disc bulging and annular fiber strains	*n* = 6	L2‐L3	L2‐L3
Wilke et al. (2012) [50]	The role of prosthesis design on segmental biomechanics: Semi‐constrained versus unconstrained prostheses and anterior versus posterior centre of rotation	*n* = 6	L2‐L5	L2‐L3 L3‐L4 L4‐L5
Wilke et al. (2013) [51]	Can prevention of a reherniation be investigated?	*n* = 7 *n* = 5	L2‐L3 L4‐L5	L2‐L3 L4‐L5
Käfer et al. (2014) [52]	Circumferential dynamic stabilization of the lumbar spine: A biomechanical analysis	*n* = 6	L4‐L5	L4‐L5
Volkheimer et al. (2017) [53]	Is pelvic fixation the only option to provide additional stability to the sacral anchorage in long lumbar instrumentation? A comparative biomechanical study of new techniques	*n* = 6	L4‐S	L4‐L5 L5‐S1
Volkheimer et al. (2018) [56]	Is intervertebral disc degeneration related to segmental instability? An evaluation with two different grading systems based on clinical imaging	*n* = 10	T12‐S1	T12‐L1 L1‐L2 L2‐L3 L3‐L4 L4‐L5 L5‐S1
Wilke et al. (2017b) [54]	Two‐piece ALIF cage optimizes the bone‐implant interface in a 360° setting	*n* = 7	L3‐S1	L4‐L5
La Barbera et al. (2018) [57]	Biomechanical advantages of supplemental accessory and satellite rods with and without interbody cages implantation for the stabilization of pedicle subtraction osteotomy	*n* = 7	T12‐S1	T12‐L1 L1‐L2 L2‐L3 L3‐L4 L4‐L5 L5‐S1
La Barbera et al. (2020) [60]	Biomechanical in vitro comparison between anterior column realignment and pedicle subtraction osteotomy for severe sagittal imbalance correction	*n* = 7	T12‐S1	T12‐L1 L1‐L2 L2‐L3 L3‐L4 L4‐L5 L5‐S1
Zengerle et al. (2020) [61]	Nucleus replacement could get a new chance with annulus closure	*n* = 1 *n* = 2 *n* = 1 *n* = 2	L2‐L3 L3‐L4 L4‐L5 L5‐S	L2‐L3 L3‐L4 L4‐L5 L5‐S1
La Barbera et al. (2021) [65]	Load‐sharing biomechanics of lumbar fixation and fusion with pedicle subtraction osteotomy	*n* = 3	T12‐S1	T12‐L1 L1‐L2 L2‐L3 L3‐L4 L4‐L5 L5‐S1
Di Pauli von Treuheim et al. (2021) [66]	Does the neutral zone quantification method matter? Efficacy of evaluating neutral zone during destabilization and restabilization in human spine implant testing	*n* = 1 *n* = 2 *n* = 1 *n* = 2	L2‐L3 L3‐L4 L4‐L5 L5‐S	L2‐L3 L3‐L4 L4‐L5 L5‐S1
Zengerle et al. (2021) [67]	In vitro model for lumbar disc herniation to investigate regenerative tissue repair approaches	*n* = 6 *n* = 2 *n* = 5 *n* = 1	L2‐L3 L3‐L4 L4‐L5 L5‐S	L2‐L3 L3‐L4 L4‐L5 L5‐S1

### 3.2 Cervical Spine

A total of *n* = 139 cervical functional spinal units had been tested under pure moment loading of 2.5 Nm for cervical spinal motion segments (C1‐T1) (Table 1). The respective range of motion and neutral zone data had been reported in 10 publications (Table 2).

### 3.3 Thoracic Spine

Overall, *n* = 129 thoracic spinal motion segments (T1‐T12) without rib cage as well as *n* = 286 with rib cage had been tested by means of pure moment loading of 5 Nm (Table 1). Range of motion and neutral zone data of the associated studies have been presented in seven publications (Table 3).

### 3.4 Lumbar Spine

Collectively, *n* = 596 lumbar functional spinal units (T12‐S) had been tested using pure moments of 7.5 Nm (Table 1). The corresponding range of motion and neutral zone data had been shown in 36 publications (Table 4).

### 3.5 Effects of Spinal Level on Spinal Flexibility

**FIGURE 3:**
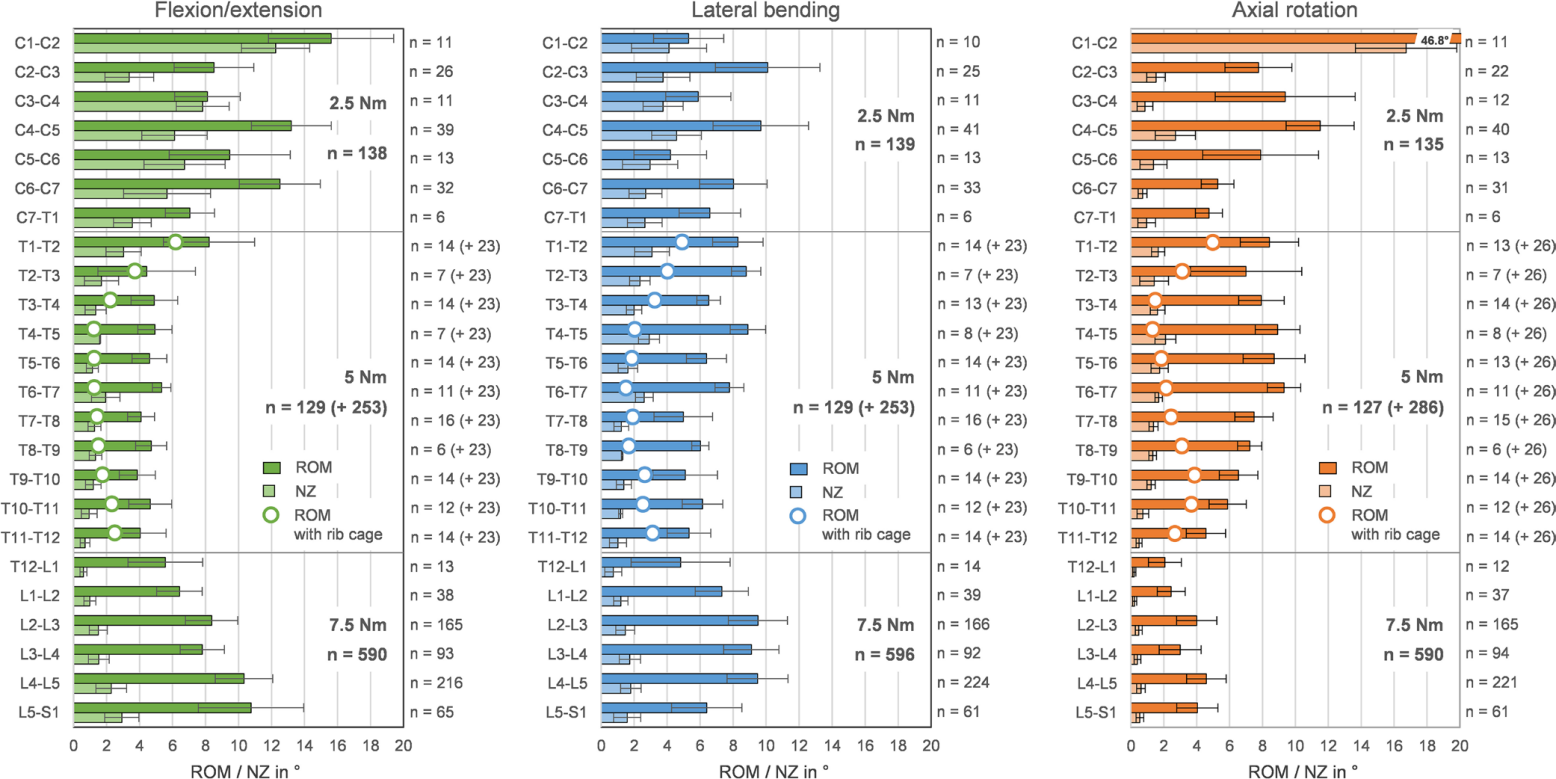
Range of motion (RoM, dark bars) and neutral zone (NZ, bright bars) data in flexion/extension, combined left and right lateral bending, and combined left and right axial rotation for all spinal levels obtained under standardized in vitro testing conditions. Data are presented as median values with median absolute deviations (MAD). Median values of thoracic spinal range of motion acquired in presence of the rib cage are depicted with framed white circles, with the respective sample sizes given in brackets.

All ranges of motion and neutral zone data acquired from testing under standardized in vitro conditions at the authors' institution were summarized as a function of spinal level in the comprehensive overview shown in Figure 3.

In flexion/extension, the range of motion and neutral zone were highest in the atlantoaxial joint (C1‐C2). Both flexibility parameters generally decreased from the mid‐cervical spine to the upper thoracic spine, were relatively even in the mid‐ and lower thoracic spine, and increased from the lower thoracic spine to the sacrum. Range of motion values of the thoracic spine were much lower when the rib cage was present.

In lateral bending, both the range of motion and neutral zone values were more equally distributed along the spine, while the highest median values were found in the upper and mid‐cervical, the upper thoracic, as well as the mid‐lumbar spine. Lowest values were also found in the mid‐thoracic spine in case the rib cage was included in the testing.

In axial rotation, by far the highest range of motion and neutral zone values were detected in the atlantoaxial joint (C1‐C2), exhibiting median values that were as high as the values of the subaxial cervical combined. Apart from that, highest flexibility values were found in the mid‐cervical and mid‐thoracic spine. However, the range of motion values of the thoracic spine decreased to a minimum in the mid‐thoracic spine with the presence of the rib cage. For the lumbar spine, both range of motion and neutral zone values were relatively low.

### 3.6 Effects of Age, Sex, and Number of Segments on Spinal Flexibility

**FIGURE 4:**
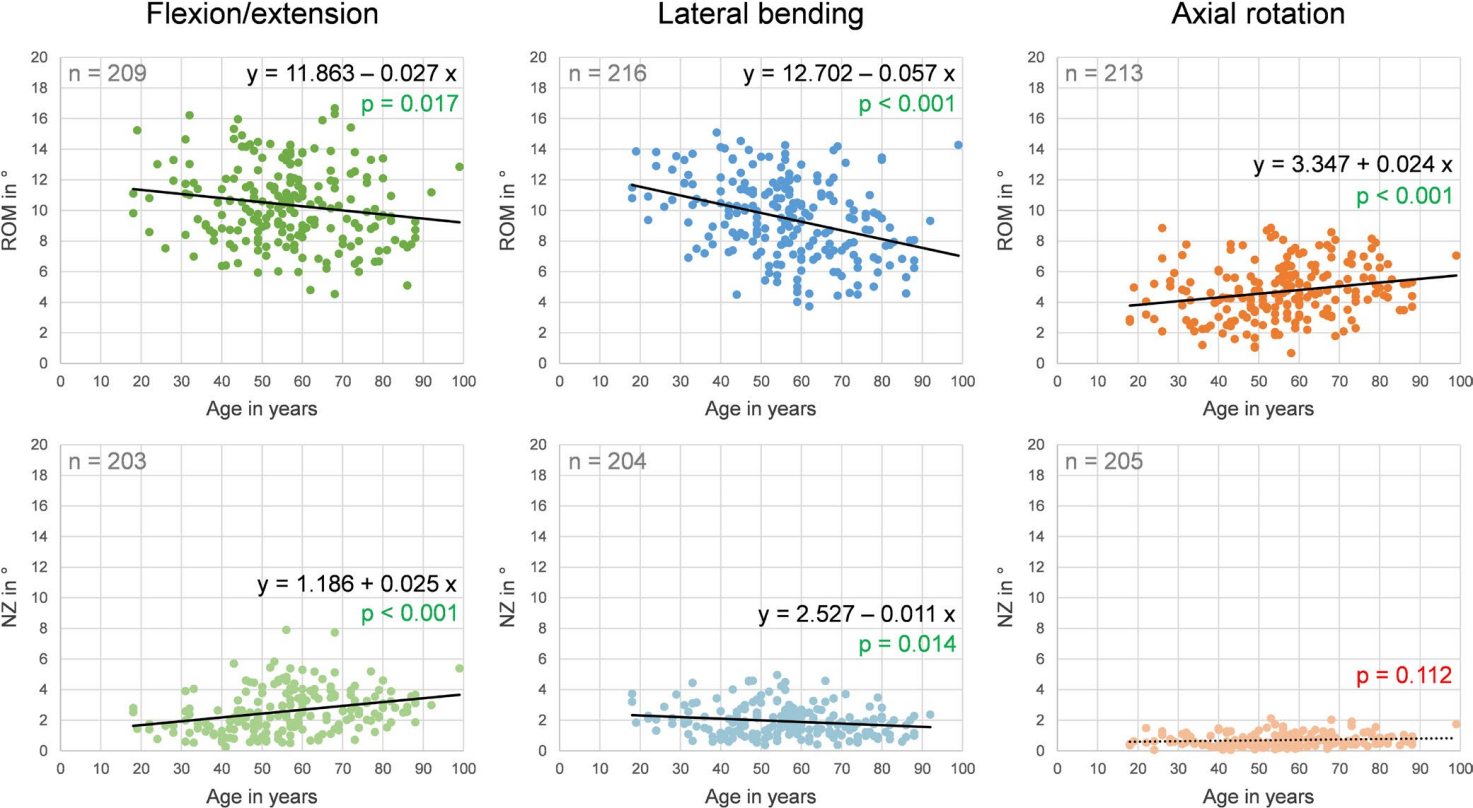
Effects of donor age on the range of motion (ROM) and neutral zone (NZ) of the segmental level L4‐L5 in flexion/extension, combined left and right lateral bending, and combined left and right axial rotation determined by linear regression analysis.

Donor age exhibited considerable effects on the range of motion and the neutral zone of the lumbar spinal level L4‐L5. While the range of motion in flexion/extension and lateral bending as well as the neutral zone in lateral bending showed significant (*p* < 0.05) negative linear regression with age, there was a significant (*p* < 0.05) positive relationship between age and the range of motion in axial rotation and the neutral zone in flexion/extension (Figure 4). Solely in axial rotation, no significant relationship between age and the neutral zone was found (*p* > 0.05). The highest effect of age was determined for the range of motion in lateral bending with a regression coefficient of −0.057, meaning that the range of motion decreased by about 0.6° per decade.

**FIGURE 5:**
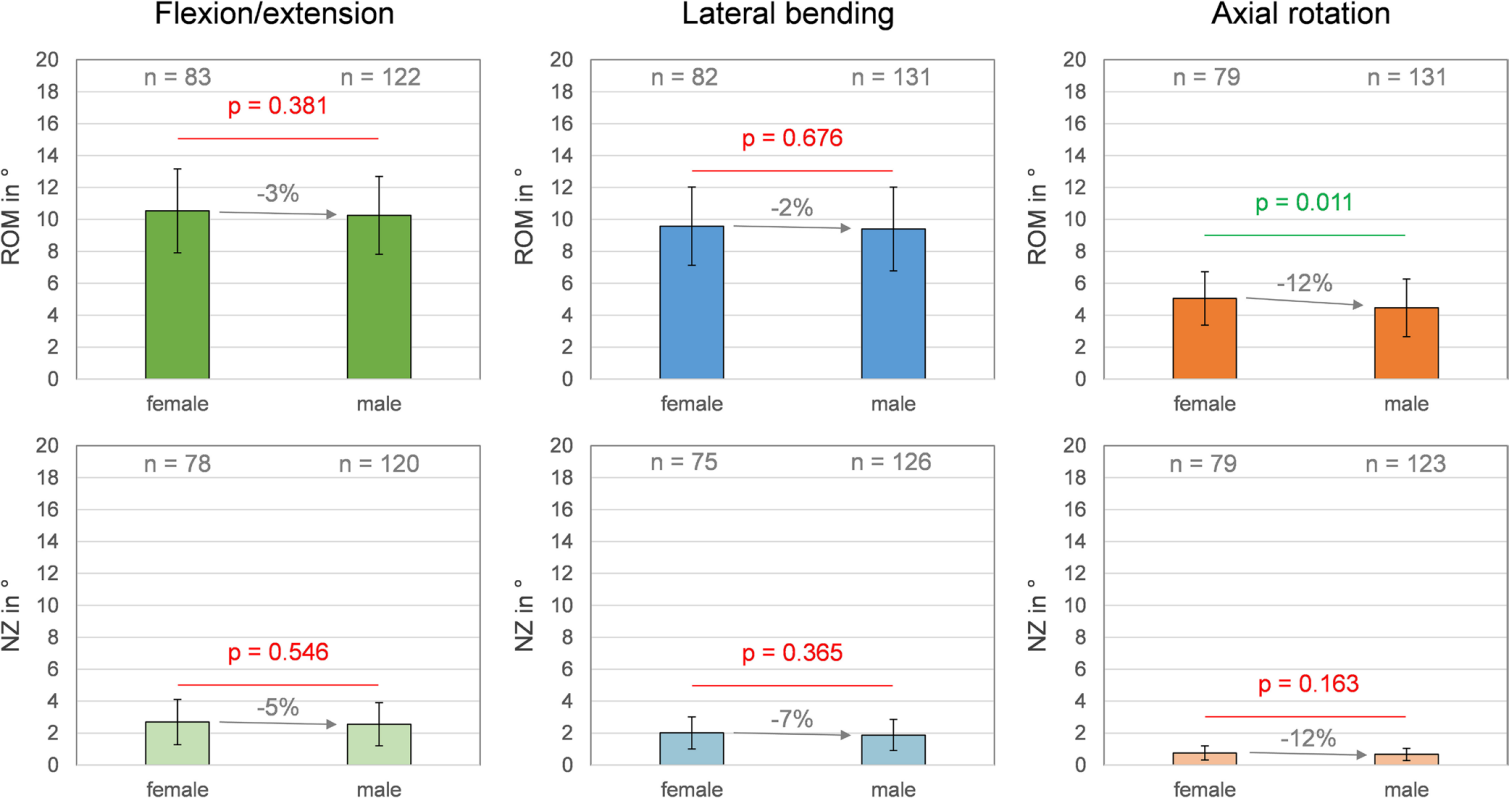
Effects of donor sex on the range of motion (ROM) and neutral zone (NZ) of the segmental level L4‐L5 in flexion/extension, combined left and right lateral bending, and combined left and right axial rotation. Data are presented as mean values with standard deviations.

Donor sex showed overall low effects on the range of motion and the neutral zone of the lumbar spinal level L4‐L5. Solely in axial rotation, a significant (*p* < 0.05) difference between specimens from female and male donors was detected, as the mean range of motion of specimens from male donors was reduced by 0.6° compared to the range of motion of specimens from female donors (−12%, Figure 5).

**FIGURE 6:**
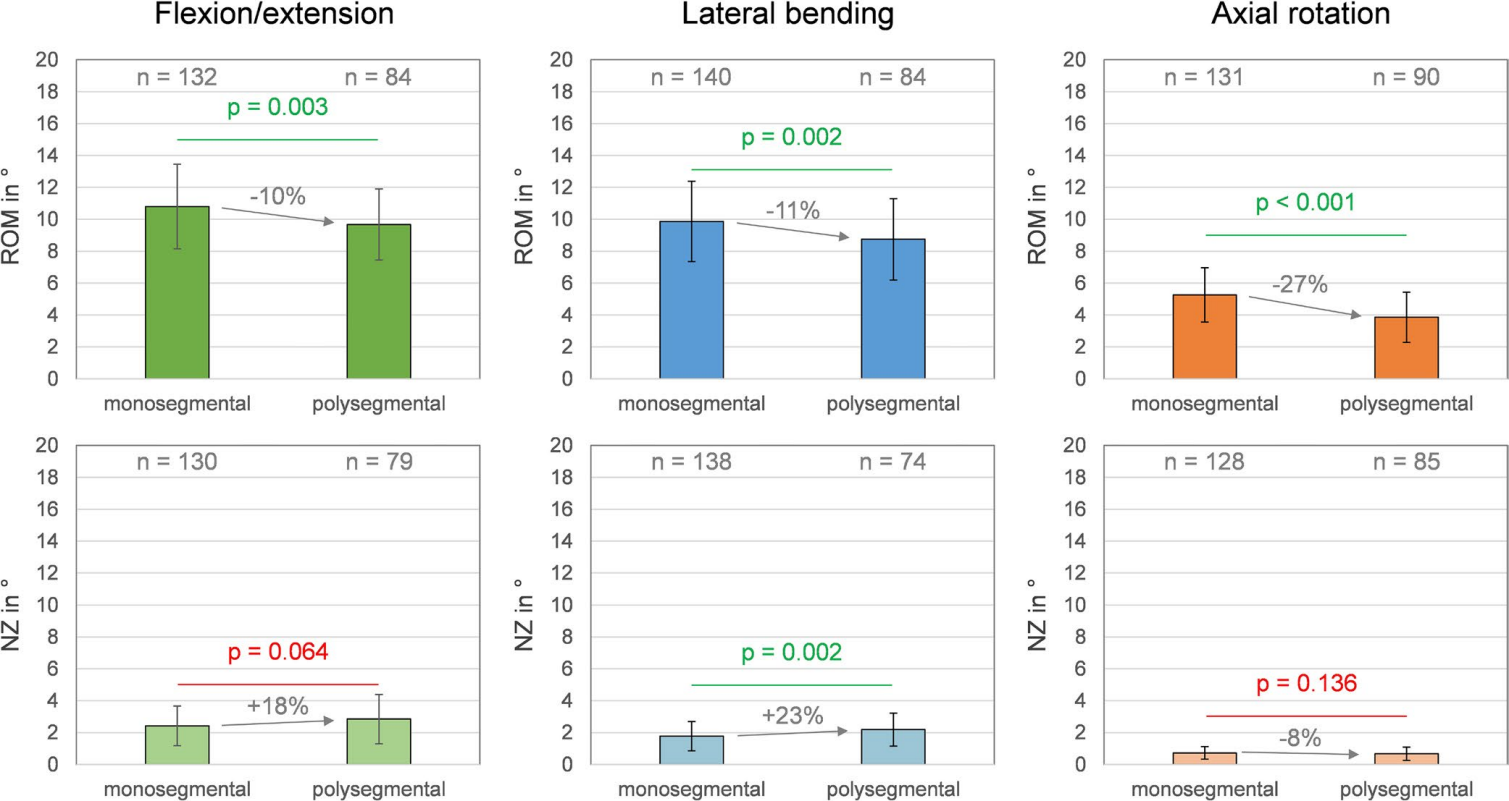
Effects of segmental length (mono‐ vs. polysegmental) on the range of motion (ROM) and neutral zone (NZ) of the segmental level L4‐L5 in flexion/extension, combined left and right lateral bending, and combined left and right axial rotation. Data are presented as mean values with standard deviations.

The number of segments of the tested specimens distinctly affected their range of motion, since for the polysegmental specimens, the range of motion was significantly (*p* < 0.05) decreased compared to monosegmental specimens, with the highest difference in axial rotation (−27%, Figure 6). The neutral zone tended to be increased for polysegmental specimens in flexion/extension and lateral bending, but significantly (*p* < 0.05) differed from monosegmental specimens solely in lateral bending.

## 4 Discussion

Representative range of motion and neutral zone data is essential for the interpretation of human spinal flexibility, for the validation of in vitro and in silico models, as well as for the development and preclinical testing of the mechanical safety and effectiveness of spinal implants. In order to provide reliable flexibility data of the spine, reproducible testing conditions are mandatory. The present work therefore aimed to give a comprehensive overview of range of motion and neutral zone data of the whole spine acquired at one single institution under standardized testing conditions with large sample sizes for the single functional spinal units.

**FIGURE 7:**
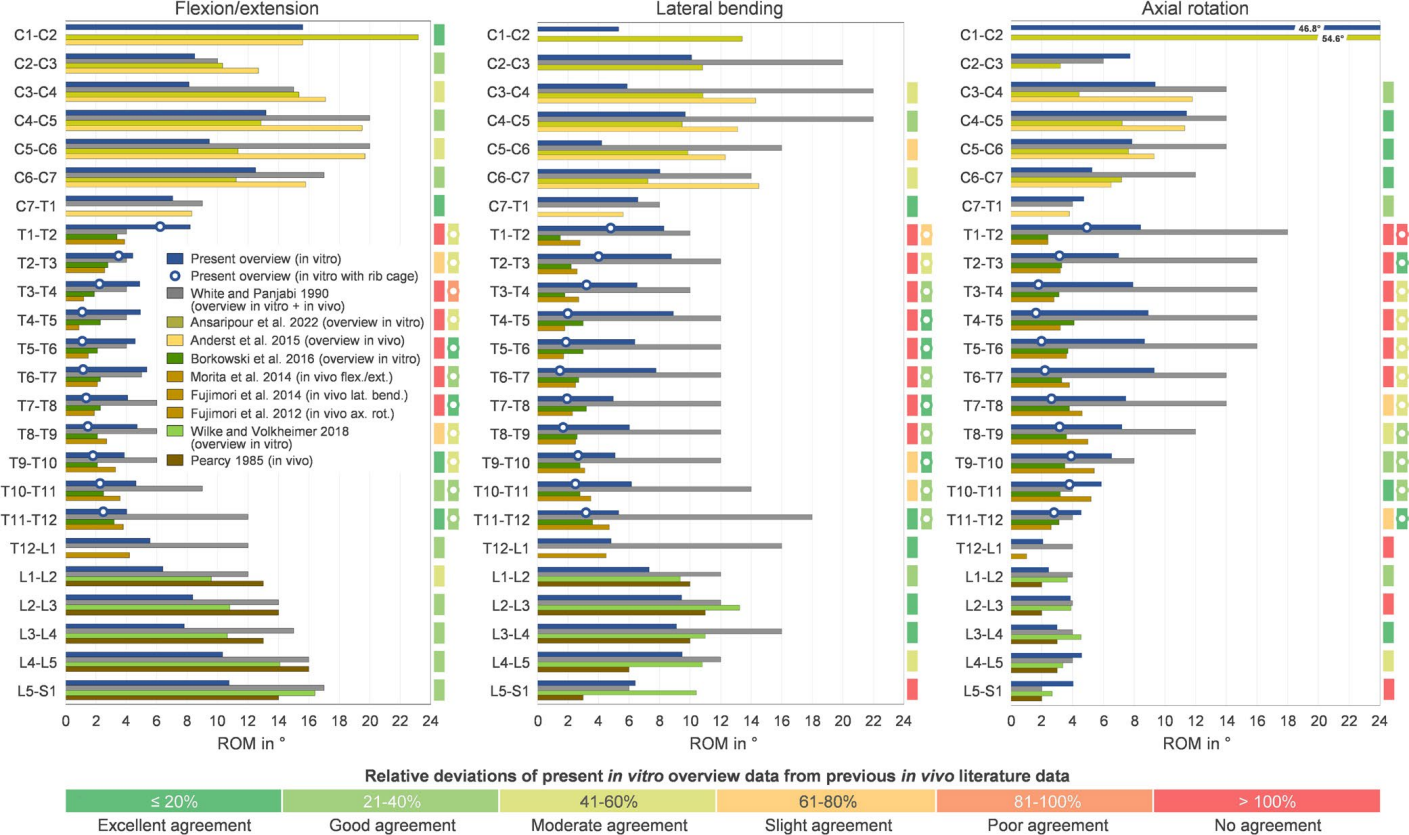
Comparison of range of motion (RoM) data of the present overview with in vitro and in vivo data from the literature. Data are displayed as median values (present overview, Ansaripour et al. 2022 [9], Borkowski et al. 2016 [8], Wilke and Volkheimer 2018 [10]), mean values (Anderst et al. 2015 [6], Morita et al. 2014 [5], Fujimori et al. 2014 [4], Fujimori et al. 2012 [3], Pearcy 1985 [2]), or representative values (White and Panjabi 1990 [14]).

Overall, the herewith presented overview of spinal range of motion data showed good agreement with previous literature, being generally in the same range with data from previous overviews of in vitro studies [8, 9, 10] as well as with data from large in vivo studies [2, 3, 4, 5, 6] for the single spinal sections (Figure 7). Particularly for the lumbar spinal levels, good to excellent agreements were found when compared to the in vivo findings of Pearcy et al. [2] in flexion/extension and lateral bending, while the range of motion tended to be higher in axial rotation. In the cervical spine, mostly moderate to excellent agreements were found, while the range of motion tended to be lower compared to the in vivo findings of Anderst et al. [6], who included very young participants in their study (20–35 years), which might explain the higher values compared to the in vitro data presented in this overview comprising a high age range (19–90 years). In contrast, the range of motion data for the thoracic spine when tested without the rib cage was distinctly higher compared to the in vivo findings of Morita et al. [5] and Fujimori et al. [3, 4], while the data overall agreed well with these in vivo studies when the rib cage was included in the experiments (Figure 7). These good agreements with in vivo data prove that the application of pure moments represents a fair compromise for the in vitro simulation of spinal loading, as it has already been shown to produce comparable flexibility [71] and kinematics [72] in prior investigations.

The data of White and Panjabi's comprehensive overview of segmental flexibility [13, 14] was found to exhibit large discrepancies from previous in vitro and vivo studies, primarily for the thoracic spinal motion segments in lateral bending and axial rotation, but also for the lower thoracic spine in flexion/extension and the upper cervical spine in lateral bending (Figure 7). In general, White and Panjabi's data tended to be higher compared to other literature data, with values being up to more than three times higher at specific thoracic spinal segmental levels compared to the data provided in the present overview without rib cage and even higher discrepancies for the entire thoracic spine when compared to data obtained with rib cage as well as compared to data from in vivo [3, 4, 5] and other in vitro [8] studies (Figure 7). White and Panjabi's overview is still considered as the benchmark for literature comparisons, as it has been widely recognized and repeatedly referenced. However, more recent overviews of single spinal sections have already started to question the representativity of White and Panjabi's data, especially in the case of the range of motion data of the thoracic spine [8], which could be confirmed by the present overview of spinal flexibility data.

Spinal flexibility data are generally subjected to several potential influencing factors, which have to be considered with regard to their interpretability and comparability. Particularly for in vitro studies, the testing conditions, such as the duration of the specimens' exposure to air and room temperature and their moisture condition, as well as accumulated load cycles and different angular deformation rates, may individually slightly but combined considerably affect the resulting flexibility of the specimens [17]. Apart from that, the loading conditions highly affect the resulting flexibility, as it is generally known that higher moments lead to higher ranges of motion, whereas additional preloads reduce ranges of motion [73, 74, 75]. For this overview, therefore, solely data acquired from testing under standardized conditions [16] were included in order to provide optimum data comparability, while the comparability between the present overview and previous overviews may be limited, as many in vitro studies from other institutions performed experiments using other testing conditions. Another major influencing factor represents the limited comparability of different specimen lengths, as findings of the present work and previous in vitro studies [76] have shown that polysegmental specimens exhibit less range of motion compared to monosegmental specimens, which might be caused by cross‐segmental ligaments providing additional stability for polysegmental specimens. Moreover, the rib cage has been shown to substantially stabilize the thoracic spine [59, 77, 78, 79], explaining the higher range of motion values found for testing without rib cage in the present overview (Figure 3) as well as in the overview of White and Panjabi [13, 14] (Figure 7). Apart from experimental conditions, morphological (disc height and diameter, facet orientations, etc.) and material properties (stiffness of anulus fibrosus, ligaments, etc.) as well as subject‐specific characteristics (age, degeneration, etc.) have to be taken into account when interpreting in vitro and in vivo data on spinal flexibility. Particularly, intervertebral disc degeneration has been determined to strongly affect the range of motion of the spine in vitro [1, 68, 80, 81, 82, 83]. Moreover, age [84, 85, 86, 87], sex [84, 86, 87], as well as intervertebral disc height [87] have been shown to be significant influencing factors on spinal flexibility. In the present work, the range of motion showed a tendency to decrease with age in flexion/extension and lateral bending, whereas it tended to increase with age in axial rotation. This corresponds well with the findings on the effects of intervertebral disc degeneration on the range of motion of the lumbar spine [82], strengthening the correlation between age and disc degeneration regarding their effects on spinal biomechanics. Sex, in contrast, exhibited solely low effects on spinal flexibility in the present work, indicating higher influences of age, disc degeneration, and morphology on spinal range of motion and neutral zone.

While the present work provides a comprehensive overview of segmental spinal flexibility in the three main motion planes with large sample sizes, some limitations of this overview have to be regarded. For instance, this overview included flexibility data from both mono‐d polysegmental testing in order to provide adequate sample sizes for each segmental level. As stated above, this included lower range of motion values for polysegmental specimens (Figure 6) and could explain discrepancies between single adjacent segmental levels, particularly in the cervical spine, where higher range of motion values were found at levels at which the proportion of monosegmental specimens was higher (Figure 3). Indeed, the sample sizes per level could have been increased to mitigate this limitation if flexibility data from experiments with non‐standard moments of the authors' institution would have been included. However, the primary aim of this overview was to provide flexibility data acquired from standardized and therefore reproducible testing conditions in order to achieve the best possible data comprehensiveness. In fact, many in vitro studies had to be performed with reduced moments depending on the study question [16], specifically in the case of testing of osteoporotic specimens [70, 88, 89] or testing including resection of functional structures [72, 77, 90]. Nevertheless, the effect of varying moments on the resulting spinal flexibility represents an important research question that will be further evaluated and separately reported by the authors. Likewise, some specimens shown in this overview were tested with moments higher than the standard in the case of worst‐case scenario testing of specific implants [91] or direct comparisons with lower spinal sections [92]. Specifically, the segmental level C1‐C2, also known as the atlantoaxial joint, which is recommended to be tested with 1 Nm, has therefore been tested with 2.5 Nm as recommended for the subaxial cervical spine [16], while the herewith presented range of motion values showed good agreement with literature data (Figure 7). Similarly, the level T12‐L1, which is anatomically seen as part of the thoracic spine, has been primarily tested with 7.5 Nm in order to provide data comparability with lumbar spinal levels. Therefore, the motion segment T12‐L1 has also been classed into the lumbar spinal section in the present overview. The testing conditions entail further limitations, such as the reduced number of loading cycles, which can be seen as practicable for experimental testing but may impede full preconditioning of the specimens, while the use of standardized testing conditions nevertheless represents a major strength of the present overview.

To conclude, a comprehensive overview of the range of motion and neutral zone data is presented. This large data collection is unique considering that all presented data have been obtained under the same, standardized loading conditions in a single testing device. While several influencing factors, such as specimen length, intervertebral disc degeneration, and the rib cage, might affect the data, this comprehensive summary of spinal flexibility data provides a detailed insight into overall spinal flexibility and represents a valid data set for the validation of in vitro studies and numerical models of the whole spine, single spinal sections, or even single segmental levels.

## Author Contributions

H.J.W. supervised all studies summarized in this work. H.J.W. and A.K. supervised this work. K.W. and C.L. collected, administered, and visualized the data. C.L. wrote the manuscript. A.K., K.W., and H.J.W. reviewed and edited the manuscript.

## Conflicts of Interest

The authors declare no conflicts of interest.

## Supporting information


Data S1.

## Data Availability

All presented data is retrievable from the Supporting Information file “RoM and NZ data.” The corresponding raw data is stored at the Institute of Orthopedic Research and Biomechanics in Ulm, Germany.
